# Supplementary material for: Differential responses of primary neuron-secreted MCP-1 and IL-9 to type 2 diabetes and Alzheimer’s disease-associated metabolites
Source: bioRxiv. 2023 Nov 17:2023.11.17.567595. Preprint. [Version 1] doi: 10.1101/2023.11.17.567595 (PMC10680853; doi:10.1101/2023.11.17.567595)
Supplement: Supplement 1 [file NIHPP2023.11.17.567595v1-supplement-1.pdf]

bioRxiv preprint doi: https://doi.org/10.1101/2023.11.17.567595; this version posted November 17, 2023. The copyright holder for this preprint (which was not certified by peer review) is the author/funder, who has granted bioRxiv a license to display the preprint in perpetuity. It is made available under aCC-BY 4.0 International license.

## SUPPLEMENTARY FIGURES

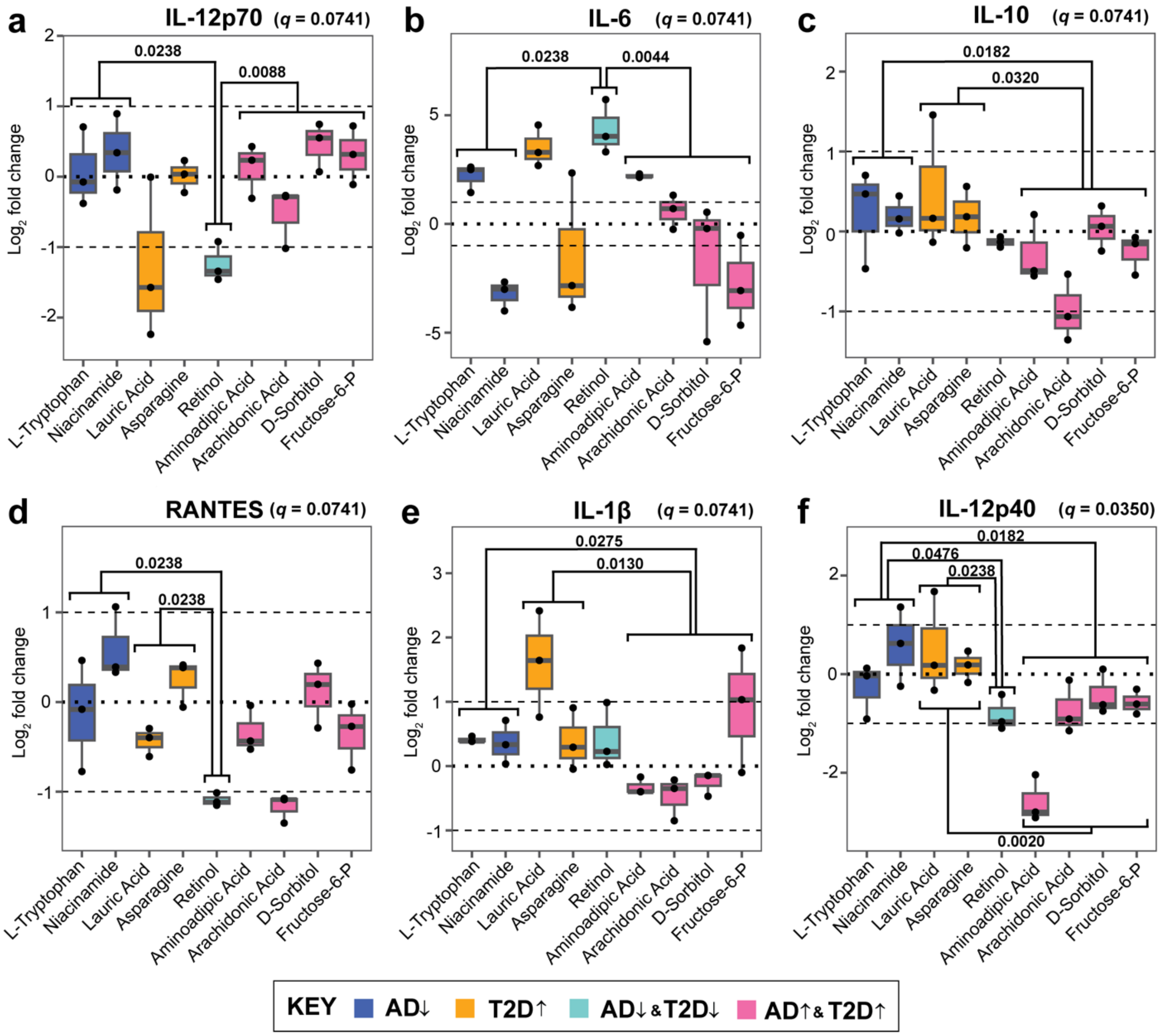


**Supplementary Figure S1. Additional Significantly Reported Results on Neurons treated with different disease associations.** The $\log_2$ ratio of cytokine concentration to vehicles that were determined significant from a Kruskal-Wallis test (FDR $q$ value next to the cytokine). Mann-Whitney pair-wise testing was applied to each metabolite group based on disease association (significance denoted within the plot). The cytokines include **(a)** IL-12p70, **(b)** IL-6, **(c)** IL-10, **(d)** RANTES, **(e)** IL-1β, and **(f)** IL-12p40.

bioRxiv preprint doi: https://doi.org/10.1101/2023.11.17.567595; this version posted November 17, 2023. The copyright holder for this preprint (which was not certified by peer review) is the author/funder, who has granted bioRxiv a license to display the preprint in perpetuity. It is made available under aCC-BY 4.0 International license.

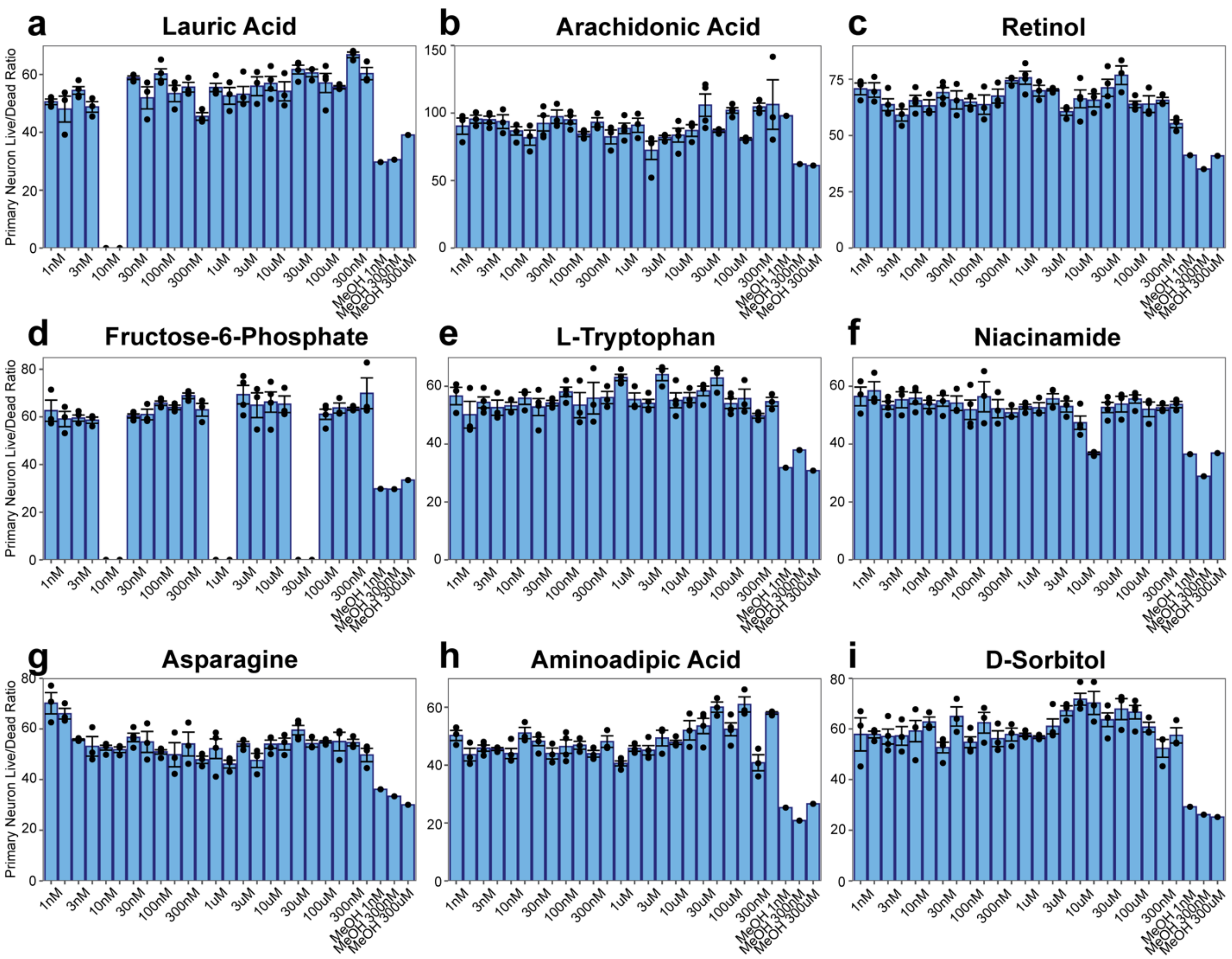


**Supplementary Figure S2. Live-dead ratio of each metabolite stimulation on primary neuron culture.** Live-dead assay was performed on **(a)** lauric acid, **(b)** arachidonic acid, **(c)** retinol, **(d)** fructose-6-phosphate, **(e)** L-tryptophan, **(f)** niacinamide, **(g)** asparagine, **(h)** aminoadipic acid, and **(i)** D-sorbitol. Per each concentration, the metabolite (left-side bar) and vehicle (right-side bar) is reported. Missing data is due to extreme outlier (lauric acid) and limitation of primary neurons (fructose-6-phosphate). Live-dead ratio is displayed as the mean □ standard error of the mean.
